# Difference Analysis Between Canine Adenovirus Types 1 And 2

**DOI:** 10.3389/fcimb.2022.854876

**Published:** 2022-03-11

**Authors:** Yanzhu Zhu, Jinfeng Xu, Shizhen Lian, Rui Zhang, Jinyu Hou, Minchun Wang, Xijun Yan

**Affiliations:** ^1^ Institute of Special Animal and Plant Sciences of Chinese Academy of Agricultural Sciences, Changchun, China; ^2^ College of Traditional Chinese Medicine, Jilin Agricultural University, Changchun, China

**Keywords:** infection characteristics, distinguishing methods, canine adenovirus 1, canine adenovirus 2, virus genome

## Abstract

Canine adenoviruses (CAdVs) include type 1 (CAdV-1, virulent strain) and type 2 (CAdV-2, attenuated strain). In recent years, the incidences of CAdV infections are increasing. However, they are difficult to distinguish when the symptoms are untypical. It is pivotal to find the differences between the two virus types for scientific, epidemiological, and specific treatment. CAdV-1 (virulent strain) and CAdV-2 (attenuated strain) induced canine hepatitis (ICH) and tracheobronchitis (ITB), respectively, but the clinical symptom is not obvious. CAdV-1 and CAdV-2 have the same genome structure, diameter, morphological features, and cytopathic features, but the same character hinder the diagnose time of the serotypes. CAdV-1 and CAdV-2 have a difference in the genome sequence, coding proteins, viral activity, hemagglutination patterns. After infection, pathogenicity and transmission route are different between the two serotypes. Sequence alignment, PCR, Real time-PCR assay are useful methods to distinguish the two serotypes. The attenuated live CAdV-2 vaccine is currently used to protect against CAdV-1, but it also has a risk. The further research should focus on the pathogenicity mechanism and the useful vaccine for the two serotypes of canine adenovirus.

## Introduction

Canine adenovirus (CAdV) belongs to the *Adenoviridae* family and *Mastadenovirus* genus (Decaro et al., 2008). CAdV is classified into canine adenovirus type 1 (CAdV-1) and type 2 (CAdV-2) serotypes (Decaro et al., 2008). Infectious canine hepatitis (ICH) induced by CAdV-1 is characterized by acute hepatitis. Infectious tracheobronchitis (ITB) induced by CAdV-2 is characterized by respiratory symptoms (Balboni et al., 2015). There is a high frequency of CAdV-1 and CAdV-2 coinfection (Balboni et al., 2014; Crespo et al., 2019; Headley et al., 2019a). Thus, the diagnosis and distinction between CAdV-1 and CAdV-2 are important for scientific, epidemiological, and specific treatment in domestic dogs and wildlife.

The differences between CAdV-1 and CAdV-2 have been reviewed by Marusyk (Marusyk et al., 1970; Marusyk, 1972; Marusyk and Hammarskjold, 1972), Hamelin (Hamelin et al., 1984), Zhong (Zhong and Yang, 1990), Linné (Linné, 1992) and Decaro (Decaro et al., 2008). Accordingly, it is necessary to update the currently available literature on the differences between CAdV-1 and CAdV-2 as the related research has been developed for nearly 20 years. Clear differentiation between CAdV-1 and CAdV-2 will help appropriate diagnosis and may prevent the further spreading of CAdVs among domestic dogs and wildlife. Therefore, the virus genome, viral activity, infection characteristic, distinguishing method and vaccination development of CAdV-1 and CAdV-2 are evaluated.

## Virus Genome

**Figure 1:**
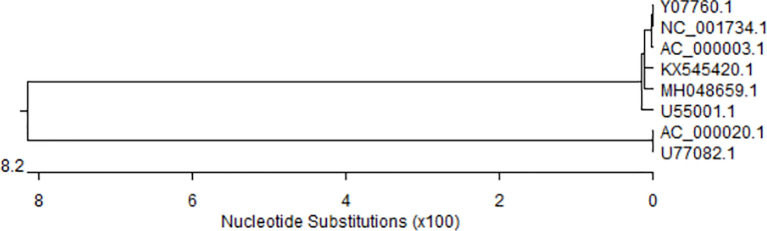
Genetic elolusion analysis with MEGA 5. CAdV-1 (AC_000003.1, U55001.1, Y07760, NC_001734.1, MH048659, KX545420.1,. CAdV-2 (AC_000020.1, U77082.1).

**Figure 2:**
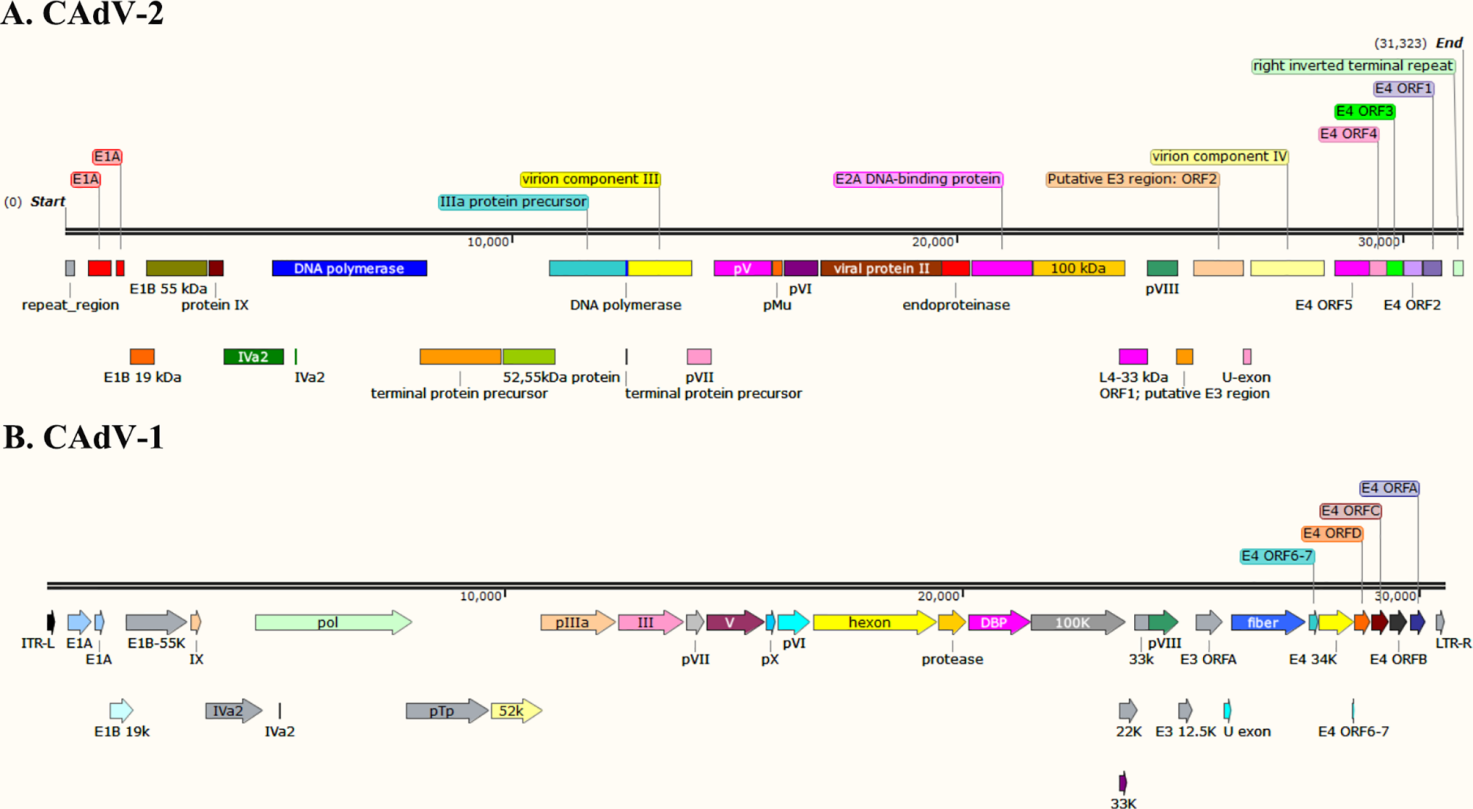
The genome maps of the CAdV-1 and CAdV-2. **(A)** CAdV-2, **(B)** CAdV-1.

The whole genome of CAdV-1 and CAdV-2 have been uploaded to the GeneBank (Shibata et al., 1989; Morrison et al., 1997; Zhu et al., 2021). The genetic evolution tree of the virus is shown in **Figure 1**. The genome maps of CAdV-1 and CAdV-2 are showed in the **Figure 2**. The Inverted terminal repeats (ITR) are vital in the replication of the CAdV-1 and CAdV-2, and the ITR length of CAdV-1 (AC_000003.1, 161bp) and CAdV-2 (M17111.1, 196 bp) are different. Cell transformation is a multistep process regulated by the cooperation of several adenoviral gene products encoded in the early regions 1 (E1) and 4 (E4) (Ip and Dobner, 2020). The E1 can participate in cell transformation and promote/inhibit the expression of cellular and viral genes. The E1 region of CAdV-2 has the complementary function of complete cell transformation, but that of CAdV-2 does not have the function (Shibata et al., 1989). The homology between the E1 region of CAdV-1 and CAdV-2 is 75% (Spibey et al., 1989). The region between E1 to the end coding region of the first protein in E1B is 153 bp longer in CAdV-2 than that in CAdV-1. E3 is a nonessential region for adenovirus replication, but E3 gene products play an important role in inhibiting host antiviral immune defense in vivo. Genes on both ends (fiber and pVIII) of the E3 region of CAdV-1 and CAdV-2 show high homology. The E3 region of CAdV-2 (U77082.1, 31323) is approximately 500 bp longer than that of CAdV-1 (AC_000003.1, 30536bp). The 500 bp insertion of the E3 region contributes to the observed biological differences between Cav-1 and Cav-2. CAdV-1 infection was found in the Eurasian wolf (Canis lupus lupus) with CAdV-2 vaccine in a French zoological park (Dowgier et al., 2018). The present of CAdV-1 infection suddenly occurs without epidemiology evidence, and it indicates that the sequential circulation of canine adenoviruses 1 and 2 may occur.

Overall, 75% homology is found, and the sequence alignment can be used to distinguish CAdV-1 and CAdV-2.

## Virus Activity

### Viral Structure

CAdV-1 and CAdV-2 have typical morphological features when observed under an electron microscope. The diameter of CAdV is approximately about 70-90 nm, and the shape is icosahedral (Ramidi et al., 2019). The linear double-stranded DNA is wrapped in a protein shell (Verin et al., 2019). The capsid consists of 252 shell grains, of which 240 are hexons, which make up the surface of the icosahedron. The other 12 are penton bases, which are located on the top of the icosahedron. Each has a fiber extending outwards, and the top of the fiber has a ball, namely the Knob. The Knob has the function of adsorbing cells and agglutinating erythrocytes (Yang et al., 2019a). The sialic acid has a binding site on the top of the CAdV-2 knob (Seiradake et al., 2009b). Crystal structures of CAdVs have been reported (Seiradake et al., 2009a), and the three-dimensional structure of CAdV-2 capsid has been constructed. The capsid is responsible for tissue tropism, and the capsid of the CAdV-2 is modified to alter tissue tropism (from canine to human) compared with human adenovirus (hAdV)-5 (Schoehn et al., 2008). However, the three-dimensional structure of CAdV-1 capsid has not been constructed.

Both CAdV-1 and CAdV-2 contain soluble components such as hexon and penton base (Zhong and Yang, 1990). Moreover, hexon is found to carry the complement binding reaction antigen common to the mammalian adenovirus group, and penton base is found to be a completely soluble hemagglutinin (Zhong and Yang, 1990). However, the binding characteristics of the two serotypes for hexon are significantly different because of the structural difference of hexon (Zhong and Yang, 1990). The fiber diameter of CAdV-2 is approximately 35 ~ 37 nm, and it is 10 nm longer than that of CAdV-1. The fiber plays a decisive role in the whole process of the binding of the adenovirus to a host cell receptor. Further, 22 and 40.7 kDa polypeptide are encoded by the E3 regions of CAdV-1 and CAdV-2, respectively (Linné, 1992; Qin, 2013). These two polypeptides share conserved amino and carboxyl domains, and show conservation at the nucleotide level.

Therefore, they share the same crystal structures and soluble components in the icosahedral. They have differences in the genome structure, diameter, morphological features, encoding proteins (knob, fiber, hexon, and E3 protein) (**Figure 2**).

### Culture Characteristics

The two serotypes of CAdVs have frequently been proliferated in MDCK cells (Carinhas et al., 2016; Pizzurro et al., 2017). The CAdV-1 can grow and proliferate in the testicular, lung, liver, and spleen cells of dogs. CAdV-1 can also proliferate in cells of other members of the canine family, such as raccoon dog kidney cells. The cells of other animals far related to dogs are also sensitive to CAdV-1, e.g., as the primary cells of pigs, guinea pigs, hamsters, minks, and other animals (Zhong and Yang, 1990). In contrast, CAdV-2 can only infect tissue cells from the dogs (Fan et al., 2009). CAdV-2 is not sensitive to primary or passage cells of other animals (such as humans, sheep, monkeys) (Fu et al., 2004).

They show the same cytopathogenic effects (grape-like cytopathic changes) on cell culture (Fan et al., 2009; Yang et al., 2010). However, the viral arrangement in the infected cell is different. CAdV-2 is closely arranged in a typical crystalline structure in the nucleus of the infected cells (Fan et al., 2009). The arrangement of the CAdV-1 is relatively loose, and the crystals formed are not as obvious as those seen in CAdV-2 (Yang et al., 2010). Further, CAdV-1 releases progeny virus through nuclear membrane degeneration, whereas CAdV-2 releases progeny virus through nuclear membrane budding (Zhong and Yang, 1990).

Therefore, they are different in the culture cell types.

### Hemagglutination and Antigenicity

Luo proposes that the relationship between the two serotypes of CAdV is mainly a one-way relationship because CAdV-1 contains more common antigenic determinants compared with CAdV-2 (Luo et al., 2008). Whetstone et al. have also reported that the two serotypes have cross-reactions (Whetstone, 1988). The serological analysis shows similarities and differences between CAdV-1 and CAdV-2. An anti-serum of CAdV-2 can neutralize the infectivity of a homologous virus (CAdV-1) and a heterologous virus (CAdV-2) with the same ability. However, the anti-serum of CAdV-1 can only neutralize the homologous virus (CAdV-1). Furthermore, Accordingly, CAdV types are distinguishable based on the antigenicity. Because CAdV-1 and CAdV-2 have cross-protection, live attenuated CAdV-2 vaccines are often used to protect animals from CAdVs.

CAdV-1 and CAdV-2 show different hemagglutination patterns (Marusyk and Yamamoto, 1971). CAdV-2 agglutinate chickens, human (O-type), and albino rat erythrocytes, but cannot agglutinate erythrocytes of mice, geese, pigeons, ducks, cattle, sheep, pigs, etc (Fan et al., 2009). In contrast, CAdV-l can agglutinate the erythrocytes of humans (O-type), albino rats (Yang et al., 2010), guinea pigs, and chickens (Hongmei Guo and Ruiliang, 2010). This difference is associated with different fiber structures between the two serotypes. The Knob of the CAdV has the function of adsorbing and agglutinating erythrocytes (Yang et al., 2019a). Their hemagglutinins bind to different receptors on the surfaces of erythrocytes.

Further, the properties of hemagglutinin receptor complexes are different. CAV-2 binding depends on electrostatic interactions, sialic acid-binding, and the coxsackievirus and adenovirus receptor (CAR) on human erythrocytes (Seiradake et al., 2009b). Based on this characteristic, we can distinguish the two types of CAdVs (Luo et al., 2008). However, it is not a regular detection method for CAdVs because dogs are commonly vaccinated with live attenuated CAdV-2 vaccines. The serum antibody of the CAdV-2 will present in most vaccine dogs and exert hemagglutination patterns. It will perturb the diagnosis of the CAdV-2 through hemagglutination patterns. Besides, CAdV-1 and CAdV-2 coinfection are often observed.

Therefore, CAdV-1 and CAdV-2 have cross-reactions and display different hemagglutination patterns. But an unambiguous diagnosis is not possible by hemagglutination test if there is coinfection with CAdV-1 and -2.

## Infection Characteristics

### Pathogenicity

The two serotypes differ in their pathogenicity, and this may be attributed to the fact that CAdV-1 and CAdV-2 are virulent and attenuated virus strains, respectively. Thus, CAdV types are distinguishable by pathogenetic characteristics. The clinical symptoms of CAdV-1 have been observed in the fennec fox (Vulpes zerda) (Choi et al., 2014), red fox (Vulpes vulpes) (Walker et al., 2016a), gray fox (Urocyon cinereoargenteus) (Gerhold et al., 2007), and arctic fox (Vulpes lagopus) (Balboni et al., 2019). However, CAdV-1 replicates in healthy red foxes (Vulpes vulpes) without clinical symptoms (Walker et al., 2016b), but it does not always have to be asymptomatic. The susceptibility of a silver fox to CAdV-1 is higher than that of the arctic foxes, and that of the arctic foxes is higher than that of dogs (Sun et al., 2019). Based on the recent data (Balboni et al., 2013; Walker et al., 2016b; Hechinger et al., 2017), red foxes show low susceptibility to CAdV-1, but the typical lesions of CAdV-1 are rare (Thompson et al., 2010b; Walker et al., 2016a). The rare typical lesions may attribute to the cross-protection conferred by earlier CAdV-2 infection, leading to milder clinical outcomes (or subclinical course) of CAdV-1 infections. In general, the potential influence of existing immunity toward CAdV-2 may also interfere with the clinical outcome of CAdV-1 infection. The pathogenicity of CAdV-1 F1301 strain in dogs, arctic foxes, and silver foxes is different (Sun et al., 2019). This indicates that the pathogenicity of CAdV-1 is different in different fox species.

Infection with CAdV-1 is also accompanied by other pathogenic infections. Fungal infection is found in a dog coinfected with CAdV-1, canine parvovirus type 2 (CPV-2), and Cladosporium halotolerans (Headley et al., 2019a). Further, coinfection of pasteurella pneumotropica and CAdV-1 is reported in a puppy (Pintore et al., 2016). CPV and CAdV-1 co-infection has been observed in 51% of wolves as a previous study (Millán et al., 2016). Co-infection with another pathogen can exacerbate CAdV-1 infection, increasing the mortality rates. The clinical signs of CAdV-2 infections are rarely apparent. The typical clinical symptom of CAdV-2 are observed in CPV-2 (Silva et al., 2014), canine distemper virus (Chvala et al., 2007), and mycoplasma cynos (Chvala et al., 2007) infection animals. Co-infection with other viruses and bacterial exacerbates CAdV-2 infection.

In brief, CAdV-1 and CAdV-2 are virulent and attenuated strains, respectively. Co-infection with other pathogens enhances the pathogenicity of the two serotypes of CAdVs. Further, the pathogenicity of CAdV-1 from different fox species is different.

### The Host Spectrum

The host spectrum of CAdV-1 is extensive. CAdV-1 is found in dogs (Chouinard et al., 1998; Balboni et al., 2014), foxes (Gerhold et al., 2007; Balboni et al., 2013; Thompson et al., 2013; Choi et al., 2014), wolves (Millán et al., 2016), raccoons (Hechinger et al., 2017), Eurasian River Otter (Lutra lutra) (Park et al., 2007), and coyotes (Canis latrans) (Miller et al., 2009), brown bears (Ursus arctos) (Ramey et al., 2018). The spectrum is believed to be narrow before, but new findings suggest that these serotypes also have a vast spectrum. Infection with CAdV-2 has been documented in dogs (Gerhold et al., 2007; Balboni et al., 2014; Millán et al., 2016; Tİmurkan et al., 2018; Yang et al., 2019b), Marsican brown bears (Ursus arctos marsicanus) (Di Francesco et al., 2015), raccoon (Dowgier et al., 2018), raccoon dogs, dogs, horses, cattle, cats (Yang et al., 2018) and wolves (Millán et al., 2016). Further, CAdV-2 is present in healthy red foxes, indicating that CAdV-2 shows subclinical circulation in wild carnivores (Balboni et al., 2013). Other evidence also shows the prevalence of CAdV-2 in the wildlife of zoological parks, given that animals with CAdV-2 infection did not show typical clinical symptoms. Another study that conducted a serological evaluation of ill or dead wild animals reported widespread CAdV-1 exposure in wildlife from different countries (Dowgier et al., 2018).

Wild foxes are considered to be the bridge between domestic animals and wildlife in terms of CAdV-1 epidemiology. Based on serological studies, CAdV is found to be circulating in 97% of island foxes (Urocyon littoralis) in California, (Garcelon et al., 1992), 88% of grey foxes in California (Riley et al., 2004), and 94.7% of wolves (Canis lupus) in Alaska (Stephenson et al., 1982). The seroprevalence of CAdV in red foxes has been reported to be 19% -64.4% in the UK (Thompson et al., 2010b; Walker et al., 2016b), 3.5% in Germany (Truyen et al., 1998), 59.6% in Scandinavia (Akerstedt et al., 2010), and 23.2% in Australia (Robinson et al., 2005).

The red foxes are a source for transmitting CAdV-1 infection to other species (Verin et al., 2019a). Canine adenovirus type-2 infections are found in neotropical otters (Lontra longicaudis) from southern Brazil (de Mello Zanim Michelazzo et al., 2022). Red foxes have more chances to contact domestic dogs because red foxes are extensively distributed in Europe and have an intrusive behavior (Bateman and Fleming, 2012). This results in a growing concern regarding the threat of CAdV infection from livestock to wildlife. In recent days, further evidence is necessary to ascertain transmission in carnivore species susceptible to CAdV. More attention should be paid to CAdV infection dynamics in wildlife.

Thus, the host spectrum of CAdV-1 and CAdV-2 is extensive. Red foxes, pine martens, and otters, with inappropriate infections, are the sources of CAdV-1.

### Clinical Symptom

CAdV-1 and CAdV-2 have different tissue tropism (Buonavoglia and Martella, 2007). CAdV-1 are identified within the epithelial cells of the liver and lungs (Headley et al., 2019). The CAdV-2 mainly invades the respiratory tract epithelium and intestinal epithelium (Michelazzo et al., 2020).

CAdV-1 transmit through feces, saliva, respiratory secretions, and urine, but CAdV-2 has an oronasal transmission (Decaro et al., 2008). The main manifestations of CAdV-1 in dogs, coyotes, raccoons, opossum rats, and skunks are hepatitis, and that in foxes and black bears is encephalitis (Fan et al., 1992; Laurenson et al., 1997), but hepatitis has also been well documented as a symptom of infection in foxes (Thompson et al., 2010a). Neurologic signs of CAdV-1 are observed occasionally in dogs and may be attributed to the damage of the blood vessels in the nervous system (Hornsey et al., 2019). The deposition of immune complexes may induce iridocyclitis (blue eye) and interstitial nephritis after recovery from CAdV-1 infection (Decaro et al., 2008). CAdV-1 can also cause simple respiratory diseases (Hornsey et al., 2019). Low titers of CAdV-2 have been detected in lung tissue samples (Sun et al., 2019). CAdV-2, however, causes infectious laryngotracheitis, pharyngitis, necrotizing bronchitis, and other respiratory diseases in dogs and foxes (Balboni et al., 2015a). However, fever is rare in infections with CAdV-2. Once the disease occurs, it is difficult to treat, and the mortality rate is high.

CAdV-1 and CAdV-2 induce different clinical symptoms. As CAdV-1 is also detected in the lung, respiratory symptoms are not included as an index to diagnose CAdV-1 and CAdV-2.

### Histopathology

The histopathological changes in the Kupffer’s cells and hepatocytes of dogs livers with CAdV-1 include swelling, yellowish color, centrilobular necrosis, neutrophilic and mononuclear cell infiltration, and intranuclear inclusions (Decaro et al., 2008). Hepatocytes with CAdV-1 are vacuolated, and cell necrosis is rare. Hepatocyte nuclei with CAdV-1 are also frequently observed to contain large basophilic inclusion bodies (Hornsey et al., 2019). The gallbladder appears thickened and edematous, and edema of the gallbladder wall maintains during infection (Decaro et al., 2008). Further, the kidney shows congestion, hemorrhage, leukocyte infiltration, and histological changes attributed to vascular damage and inflammation (Decaro et al., 2008). After recovery from ICH, interstitial nephritis and iridocyclitis may occur in dogs (Decaro et al., 2008). Congestion and hemorrhagic lesions are also observed in the spleen, lymph nodes, thymus, pancreas, and hemorrhagic enteritis (Hornsey et al., 2019).

On postmortem examination of dogs infected with CAdV-2, the histological change in the lung show red areas of consolidation necrotizing bronchitis and bronchiolitis obliterans. It attributes to the damage of bronchial epithelial cells and infection of type 2 alveolar cells (Tİmurkan et al., 2018). CAdV-2 infection results in significant respiratory illness in dogs (Yang et al., 2019b).

CAdV-1 and CAdV-2 are mainly located in the liver and lungs respectively, and they exert different histopathological changes. After infection with CAdV-1 and CAdV-2, nuclear inclusions can form in infected cells (Hornsey et al., 2019), and the formation time and the shape and type of inclusion body have their characteristics (Zhong and Yang, 1990). The liver cell swelling, degeneration, interstitial vascular congestion in the lung, and reduction of the spleen lymphocytes are observed in the silver fox, arctic fox, and dog. The positive immunohistochemical staining with CAdV-1 is observed in the liver and spleen (Sun et al., 2019).

Based on the above-mentioned data, the inclusion bodies are observed in the tissue with CAdV-1 and CAdV-2. The main histopathological changes in infections with CAdV-1 and CAdV-2 are observed in the liver and lungs, respectively.

## Distinguishing Method

In a clinical setting, ocular swabs, feces, and urine samples, and kidney, lung, and lymphoid tissues can be collected to isolate CAdV-1 and CAdV-2. Traditionally, viral isolation, electron-microscopic observation (Hongmei Guo and Ruiliang, 2010), and serological tests (Walker et al., 2016b) are used to identify CAdV-1 and CAdV-2 infections. Interestingly, respiratory secretions, trachea, and lungs can be used for CAdV-1 isolation, so the collected tissue do not indicate the type of CAdV infection. Immunofluorescence (IF) test can be used to determine CAdV in acetone-fixed tissue sections, smears, and cells. Compared with the conventional pathological methods, immunohistochemical (IHC) analysis is considered to be a reliable diagnostic method for CAdV infections. IHC of CAdV-2 diagnosis should be conducted with PCR (Yoon et al., 2010). However, the IF and IHC tests are not adequate for distinguishing between the CAdV-1 and CAdV-2, because antibodies of CAdV-1 and CAdV-2 have cross-reactions with each other. Thus, viral isolation, IF and IHC tests do not differentiate between CAdV-1 and CAdV-2. After CAdV-1 and CAdV-2 infection, nuclear inclusion body can be observed by hematoxylin-eosin (HE) staining. But HE-staining cannot identify viruses. It merely makes the intracellular inclusion bodies visible, which indicate viral infection and those inclusion bodies can give a hint as to which virus is probably involved (based on the animal involved, staining characteristics and localization of inclusion bodies within the cell), but they do not identify the virus. The recent discrimination methods are established mainly based on sequence difference. However, it is expensive to sequence the genome of the two serotypes of CAdVs. The genomes of CAdV-1 and CAdV-2 can be differentiated by restriction endonuclease analysis (Assaf et al., 1983; Hamelin et al., 1984) and DNA hybridization (Marusyk and Hammarskjold, 1972). But the method is complex and requires high purified viral DNA and more time (Decaro et al., 2008).

One pair of common primers is designed and synthesized according to the sequences of the E3 and flanking regions and a polymerase chain reaction (PCR) assay is established to amplify E3 region in CAdV-1 (508 bp) or CAdV-2 (1030 bp) (Hu et al., 2001). Furthermore, the common set of primers are used to amplify the fragments of the CAdVs in the feces and urine samples, respectively (Chaturvedi et al., 2008). This provides a useful way of identifying the nature of CAV infection. A TaqMan RT-PCR is used to determine CAdV-1 in red foxes (Vulpes vulpes) and raccoons (Procyon lotor) in Germany (Hechinger et al., 2017). Moreover, to detect and simultaneously differentiate between the two serotypes of CAdVs, a duplex RT-PCR is established based on TaqMan technology (Dowgier et al., 2016). Compared with the SYBR Green qPCR, a probe-based RT-PCR assay has high specificity. However, a real-time instrument and two specific probes are necessary to detect the different wavelengths of CAdV-1 and CAdV-2. Furthermore, probe-based RT-PCR is more expensive compared with SYBR green qPCR because probes are necessary to the primers. And nucleotide mutations will affect the specificity of reaction in probe-based RT-PCR in the hybridization sites of the probes (Balboni et al., 2015).

Melting curve analysis is also used in the SYBR Green RT-PCR for simultaneous detection and differentiation between the two serotypes of CAdVs (Balboni et al., 2015b). A binding dye is used to intercalates nonspecifically into the double-stranded DNA in SYBR^®^ Green methods, whereas the specificity is improved by sequence-specific amplification in the TaqMan^®^ approach. Accordingly, the melting curve analysis based on SYBR Green PCR is performed given that the instrumentation is less complex, the method is simple, and the procedure is cost-effective.

During PCR amplification, the double-standard DNA binds to SYBR Green, and SYBR Green is not a specific fluorescent dye. Thus, the product length and the melting curve analysis method ensured the specificity of the SYBR Green assay. Conversely, primers and probes enhance the specificity of TaqMan technology.

Therefore, sequence alignment, PCR, and RT-PCR assay are useful methods to distinguish between the two serotypes of CAdVs.

## Vaccination Development

In 1976, CAdV-1, which is cultured with MDCK, is inactivated to prepare the vaccine (Fishman and Scarnell, 1976). The inactivated CAdV-1 vaccine has immunogenicity and safety. But the inactivated CAdV-1 vaccine cannot protect the CAdV-2 from the dog, it is eliminated by the market at last. The CAdV-1 is attenuated in the MDCK by consecutive passage, the early attenuated CAdV-1 vaccine is prepared based on the attenuated virus strain (Pepevnak et al., 1960). But the attenuated virus strain has the risk of the virulence return. The attenuated CAdV-2 virus strain (Toronto A26-61) has cross-protection to CAdV-1 (Bru et al., 2010). The usage of the attenuated live CAdV-2 vaccine reduces the spread of the CAdV-1 and CAdV-2 (Bass et al., 1980). The virus shedding will find after the immunization of the attenuated live CAdV-2 vaccine, and the virus shedding will lead to the inapparent infection of the fox without CAdV-2 (Dowgier et al., 2018), and this phenomenon also attracts attention (Decaro et al., 2007). Thus, the attenuated live CAdV-2 vaccine has a risk in the clinical setting.

Therefore, the attenuated live CAdV-2 vaccine is currently used to protect against CadV-1 whereas a vaccine against CadV-1 is difficult to develop due to safety concerns and less cross-protection. However, the attenuated live CAdV-2 vaccine also has a risk.

## Conclusion

CAdV-1 (virulent strain) and CAdV-2 (attenuated strain) induced ICH and ITB, respectively, but the clinical symptom is not obvious. CAdV-1 and CAdV-2 have the same genome structure, diameter, morphological features, and cytopathic features, but the same character hinder the diagnose time of the serotypes. CAdV-1 and CAdV-2 have a difference in the genome sequence, coding proteins, viral activity, hemagglutination patterns. After infection, pathogenicity and transmission route are different between the two serotypes. Sequence alignment, PCR, RT-PCR assay are useful methods to distinguish the two serotypes. The attenuated live CAdV-2 vaccine is currently used to protect against CAdV-1, but it also has a risk. The further research should focus on the pathogenicity mechanism and the useful vaccine for the two serotypes of canine adenovirus.

## Author Contributions

YZ wrote the manuscript. JX, SL, and RZ collected the reference. JH, MW and XY review the manuscript. All authors contributed to the article and approved the submitted version.

## Funding

This work was supported by grants from the Science and technology department of Jilin Province (20200402045NC).

## Conflict of Interest

The authors declare that the research was conducted in the absence of any commercial or financial relationships that could be construed as a potential conflict of interest.

## Publisher’s Note

All claims expressed in this article are solely those of the authors and do not necessarily represent those of their affiliated organizations, or those of the publisher, the editors and the reviewers. Any product that may be evaluated in this article, or claim that may be made by its manufacturer, is not guaranteed or endorsed by the publisher.
